# Environmental Impacts on COVID-19: Mechanisms of Increased Susceptibility

**DOI:** 10.5334/aogh.3907

**Published:** 2022-10-21

**Authors:** Stephania A. Cormier, Ayaho Yamamoto, Kirsty R. Short, Luan Vu, William A. Suk

**Affiliations:** 1 Louisiana State University, Department of Biological Sciences, and Pennington Biomedical Research Center, Baton Rouge, LA, USA; 2 The University of Queensland, Child Health Research Centre, South Brisbane, QLD, Australia; 3 The University of Queensland, School of Chemistry and Molecular Biosciences, Brisbane, QLD, Australia; 4 National Institute of Environmental Health Sciences, Superfund Research Program, 530 Davis Drive, Durham, NC, USA

**Keywords:** COVID 19, air pollution, respiratory viral infections, age

## Abstract

**Background:**

Since 2019, severe acute respiratory syndrome coronavirus 2 (SARS-CoV-2) has resulted in >554M cases and >6.3M deaths worldwide. The disease caused by SARS-CoV-2, COVID-19, has resulted in a broad range of clinical symptoms differing in severity. Initially, the elderly were identified as particularly susceptible to severe COVID-19, with children experiencing less severe disease. However, as new variants arise, the epidemiology of SARS-CoV-2 infection is changing, and the disease severity in children is increasing. While environmental impacts on COVID-19 have been described, the underlying mechanisms are poorly described.

**Objective:**

The Pacific Basin Consortium for Environment and Health (PBC) held meeting on September 16, 2021, to explore environmental impacts on infectious diseases, including COVID-19.

**Methods:**

The PBC is an international group of environmental scientists and those interested in health outcomes. The PBC met to present preliminary data and discuss the role of exposures to airborne pollutants in enhancing susceptibility to and severity of respiratory tract viral infections, including COVID-19.

**Findings:**

Analysis of the literature and data presented identified age as an important factor in vulnerability to air pollution and enhanced COVID-19 susceptibility and severity. Mechanisms involved in increasing severity of COVID-19 were discussed, and gaps in knowledge were identified.

**Conclusions:**

Exposure to particulate matter (PM) pollution enhanced morbidity and mortality to COVID-19 in a pediatric population associated with induction of oxidative stress. In addition, free radicals present on PM can induce rapid changes in the viral genome that can lead to vaccine escape, altered host susceptibility, and viral pathogenicity. Nutritional antioxidant supplements have been shown to reduce the severity of viral infections, inhibit the inflammatory cytokine storm, and boost host immunity and may be of benefit in combating COVID-19.

## Age and Susceptibility to SARS-CoV-2 Infection

Children typically experience more mild symptoms of COVID-19 when compared to adults. There is a strong body of evidence that children are found to be less susceptible to SARS-CoV-2 infection with the original Wuhan isolate. The reasons for reduced SARS-CoV-2 symptoms and infection in children remain unclear and may be influenced by a multitude of factors, including differences in target cell susceptibility and innate immune responses [1]. Using primary nasal epithelial cells from children and adults, differentiated at an air-liquid interface (ALI) we showed that SARS-CoV-2 (both the Wuhan isolate and the more recent Alpha variant) replicate to significantly lower titers in the nasal epithelial cells of children compared to those of adults [2]. This was associated with a heightened antiviral response to SARS-CoV-2 in the nasal epithelial cells of children. Importantly, influenza virus, a virus whose transmission is frequently associated with pediatric infections, replicated in both adult and pediatric nasal epithelial cells to comparable titers. We have expanded these data to show that the more recent Delta, but not Omicron variant also replicated less in children’s nasal cells [2]. Taken together, these data show that the nasal epithelium of children supports lower infection and replication of the earlier SARS-CoV-2 variants than the adult nasal epithelium. Why viral replication is increased in children with the more recent Omicron variants is not known, but it is consistent with the epidemiology showing an increased number of cases in children as these have become dominant [3]. Traffic-related air pollution exposure during childhood is associated with an increased risk of severe respiratory infections [4,5]. However, the interplay between age, environment, and COVID-19 remains unclear.

## Areas of High Particulate Pollution and COVID

As with other viruses, epidemiological data demonstrate a strong influence of environmental factors on the incidence of infection with SARS-CoV-2 and the severity of COVID-19. Areas with high particulate matter (PM) pollution have been associated with increased mortality, not only to SARS-CoV-1 but recently to SARS-CoV-2 [6,7], compared to regions with lower air pollution. The air pollution index (API), which is a simplified way to describe air quality and incorporates carbon monoxide, ozone, nitrogen dioxide, sulfur dioxide, and PM_2.5_ was used in this study; and a moderate API of 51–100 was associated with an 84% increased mortality risk. Long-term and historical exposure to elevated PM_2.5_ levels have also been associated with a significant increase in COVID mortality. Specifically, an increase of 1 µg/m^3^ in the long-term average PM_2.5_ level correlated with an 11% increase in the COVID mortality rate [7]. The increase in mortality was even greater among black individuals. These data are reviewed in more detail in a companion paper in this series [8].

**Figure 1:**
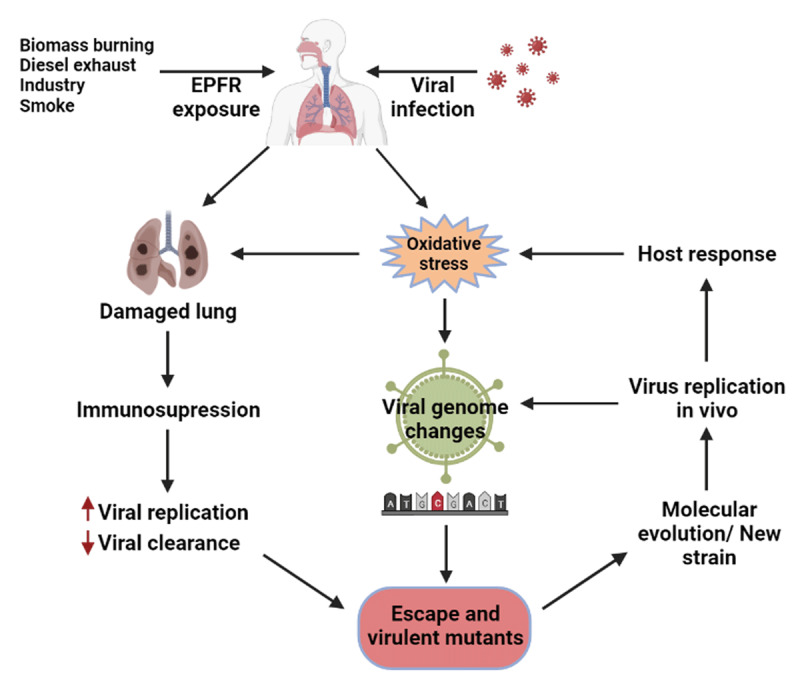
Potential roles of EPFRs in enhancing viral morbidity and mortality.

Several theories exist to explain the role of PM in enhanced morbidity and mortality of COVID-19. The first is that PM acts as a carrier for the virus - hijacking a ride on airborne PM. This has been demonstrated with other pathogens, including bacteria, fungi, and viruses [9,10,11,12], and most recently demonstrated for SARS-CoV-2 [11,13]. It has been further hypothesized that the hijacked particle could enhance viral persistence and respirability, allowing it access to the lower airways. While ambient sources of PM_2.5_ vary between locations, combustion and industrial emissions are the major producers, and PM_2.5_ from such sources typically are associated with environmentally persistent free radicals (EPFRs) [14,15]. We further posit that the presence of EPFRs on PM can: 1) damage the airways inducing an immunosuppressive pulmonary microenvironment as has been demonstrated with influenza [16,17,18]; 2) induce mutations in the viral genome, increasing infectivity and/or pathogenicity of the virus; and/or 3) oxidize surface molecules on the virus altering the ability of the immune system to recognize the virus (Figure 1).

The first two hypotheses were explored using well-differentiated human nasal epithelial cells cultured at ALI (Ethics approval: No.#UQ2017000520; HREC61894; UQ2020001742), and preliminary data presented demonstrates that exposure to moderate level of EPFRs impaired epithelial barrier and reduced mucus production. Decreasing mucus production removes part of the first-line defense of respiratory epithelial cells and would be expected to increase viral access to the cell surface receptors.

**Figure 2:**
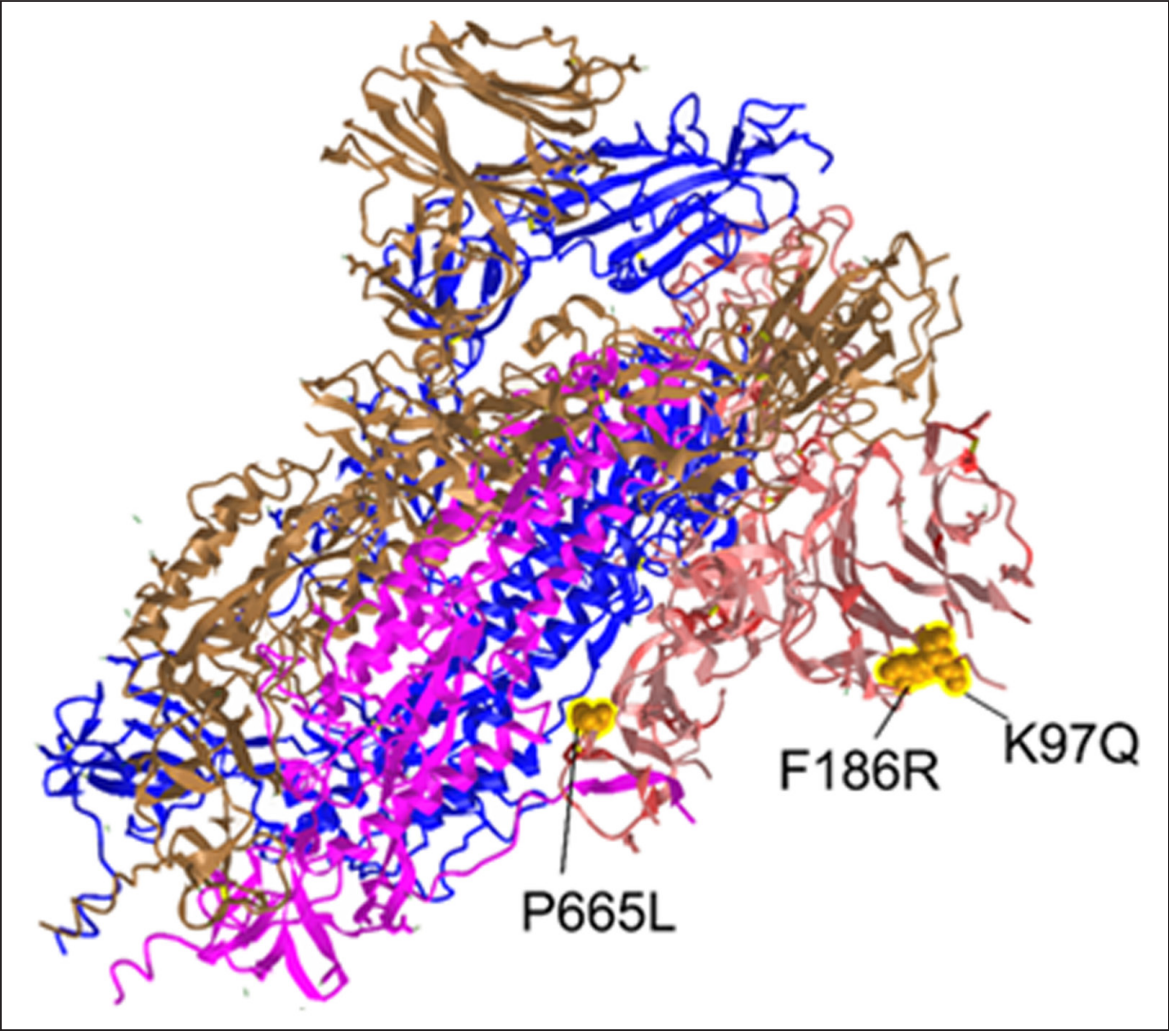
The SARS-CoV-2 NTD comprised multiple mutation at 48 and 72h post EPFR exposure. Mutations are highlighted as yellow spheres.

Supernatants containing SARS-CoV-2 viral particles from these same ALI cultures were isolated, and genetic modifications were identified by sequencing. Significant increases in the number of nucleotide changes were observed from ALIs exposed to EPFRs (i.e., a 33% increase compared to control air-exposed ALIs) for as short as 24h. While changes were observed across the genome, the largest number of changes were observed in the S gene, which codes for the Spike protein, followed by the E gene, which codes the Envelope protein. Intriguingly, mutations were observed at the N-terminal domain (NTD) of the S1 subunit and at S1/S2 cleavage site (Figure 2).

While the receptor binding domain of S1 mediates viral infection by binding to host ACE2 receptors and is recognized as the key target for neutralization antibodies, the target of NTD is still unknown [19]. Still, changes in the conformation of exposed NTD loops have been associated with increased infectivity [20].

Furthermore, many potential neutralizing antibodies targeting NTD have been identified [21,22]. These NTD-targeting antibodies target supersite epitopes harbored at the most exposed region of NTD (spanning from amino acid position 24 to 333) and have been shown to neutralize SARS-CoV-2 *in vitro* and *in vivo* [21,23,24]. Thus, our preliminary data suggest that exposure to EPFRs can alter viral infectivity and affect an immune escape. Considering the significance of pollution exposure-mediated viral respiratory diseases, we urge future studies to further investigate and empirically validate our current findings.

While it is generally recognized that alterations in the S protein could alter infectivity and impact host protection (both in terms of immune evasion and vaccine escape), the role of genetic alterations in the E protein is less understood. The E protein is important in viral assembly, budding, and pathogenesis via damage to epithelial tight junctions [25]; alterations here may thus be critical in altering morbidity.

Oxidative stress plays an important role in environmental exposure and viral infections [26,27]. When SARS-CoV-2 enters cells, nicotinamide adenine dinucleotide phosphate (NADPH) oxidase is activated, resulting in increased mitochondrial reactive oxygen species (mtROS) [28] and EPFR exposure also increases mtROS [29]. Thus, excessive production of mtROS induced by air pollution may result in oxidative stress in epithelial cells, which might be one of the key mechanisms increasing severity of COVID-19.

Infections and environmental exposure can trigger cytokine secretion as a host defense [30]. Exaggerated secretion of cytokines by both airway epithelium and immune cells such as macrophages, T cells, and neutrophils can cause organ failure and increase the severity of COVID-19 [31]. Severe COVID-19 can include ‘cytokine storm syndrome’ because of uncontrolled immune responses [32]. Both EPFR exposure and SARS-CoV-2 infection increased the pro-inflammatory cytokine, tumour necrosis factor α (TNF-α) production [29,33]. Massive accumulation of TNF-α can contribute to cytokine storm; acute lung injury, or acute respiratory distress syndrome [34,35].

PM induces oxidative stress and inflammation, and this can further enhance SARS-CoV-2-induced inflammation resulting in reduced therapeutic efficiency [24]. Multiple nutritional antioxidant supplements have been shown to reduce the severity of viral infections, inhibit the inflammatory cytokine storm, and boost host immunity [36]. Thus, those nutritional compounds may benefit COVID-19 treatment. Several clinical trials for antioxidant treatment for COVID-19 have been conducted, and some of them are still ongoing [37]. Two doses of N-acetylcysteine (NAC), a well-known antioxidant, did not decrease COVID-19 severity [38]; but a mixture of methylene blue, Vitamin C and NAC treatment increased the survival rate in severe COVID-19 patients [35]. Single antioxidant agents have not shown promising results in clinical trials. Alternative agents with better antioxidant capacity or combinations of antioxidants might be a better option for treating COVID-19.

## Conclusion

Direct evidence, in a human system, of the mechanisms by which environmental pollutants, including EPFRs increase susceptibility for respiratory tract viral infections, including COVID-19, is needed. Further, identification of methods to reduce pollutant-mediated susceptibility using readily available therapies will be of great benefit. The significance of some of the data presented comes from the potential to reduce susceptibility to respiratory tract viral infections in the billions of people globally who are exposed to EPFRs from combustion and industrial processes. Finally, it is anticipated that resulting data will be essential to guide policy related to future pandemics.

## Abbreviations

ALI – air-liquid interface

API – air pollution index

PM – Particulate Matter

EPFR – Environmentally persistent free radicals

NAC – N-acetylcysteine

ROS – reactive oxygen species

SARS-CoV-2 – severe acute respiratory syndrome coronavirus 2

TNF-α – tumour necrosis factor α
